# Acceleration of Puberty During Growth Hormone Therapy in a Child with Septo-Optic Dysplasia

**DOI:** 10.4274/Jcrpe.1187

**Published:** 2014-06-05

**Authors:** Gönül Çatlı, Ayça Altıncık, Ahmet Anık, Korcan Demir, Handan Güleryüz, Ayhan Abacı, Ece Ece Böber

**Affiliations:** 1 Dokuz Eylül University Faculty of Medicine, Department of Pediatric Endocrinology, İzmir, Turkey; 2 Dokuz Eylül University Faculty of Medicine, Department of Radiology, İzmir, Turkey

**Keywords:** Septo-optic dysplasia, hypopituitarism, growth hormone, Precocious puberty

## Abstract

Septo-optic dysplasia (SOD) is a heterogeneous disorder of the central nervous system characterized by various endocrinological and neurological findings. It is a complex disease caused by a combination of genetic and environmental factors. Herein, we report the case of a 5.5-year-old girl who presented with short stature and strabismus. Ophthalmological examination revealed bilateral optic nerve hypoplasia. Ectopic posterior pituitary and bilateral optic hypoplasia were detected on brain magnetic resonance imaging. The presence of bilateral optic nerve hypoplasia and hypopituitarism led to the diagnosis of SOD. An abated growth hormone (GH) response was found in the GH stimulation test and GH replacement therapy was initiated. At the end of the first year of clinical follow-up, secondary hypothyroidism was detected and L-thyroxine was added to the treatment. At the age of 8.25 years, thelarche was noted and 6 months later, the patient presented with menarche. At this time, the bone age was 12 years and the basal luteinizing hormone level was 7 mIU/mL. These findings indicated acceleration in the process of pubertal development. We report this case (i) to emphasize the need to investigate hypopituitarism in cases with bilateral optic nerve hypoplasia and (ii) to draw attention to the fact that during the follow-up of SOD cases receiving GH therapy, inappropriate acceleration of growth velocity and rapid improvement in bone age may be predictive of central precocious puberty development.

## INTRODUCTION

Septo-optic dysplasia (SOD) is a heterogeneous disorder characterized by midline brain defects and pituitary hormone deficiencies. In 1941, Reeves first reported the association of optic nerve and septum pellucidum abnormalities (1). Fifteen years later, the clinical signs observed in SOD were defined as De Morsier syndrome. In 1970, hypophyseal dysfunction was shown in patients with SOD (2). The incidence of this disease, which is equally seen in both sexes, is 1/10 000 (2,3,4). Diagnosis is made by the presence of two out of three criteria, namely, optic nerve hypoplasia, hypopituitarism and midline brain defects (septum pellucidum and/or corpus callosum agenesis/hypoplasia) (2,3,4,5). SOD has a multifactorial etiopathogenesis and it has been shown that mutations in HESX1, SOX2, SOX3 and OTX2 genes are responsible for less than 1% of cases (2,3,4). Although many cases are sporadic, the frequency of autosomal recessive inheritance is increased especially in consanguineous marriages. Autosomal dominantly inherited cases have also been reported (2). SOD is more commonly encountered in preterm babies and in babies of young, diabetic or primigravida mothers (2,3,4).

In the present report, a 5.5-year-old girl diagnosed to have SOD is presented. The patient was found to have optic nerve hypoplasia, cranial magnetic resonance imaging (MRI) findings, growth hormone (GH) deficiency, central hypothyroidism and at a later date, developed central precocious puberty (CPP), a disorder which is rarely reported to accompany SOD. 

## CASE REPORT

**Figure 1:**
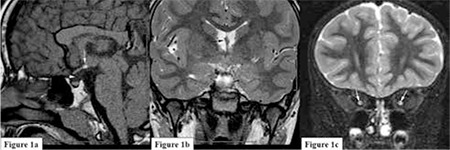
In the T1-weighted sagittal section; a) ectopic neuro-hypophysis (white arrow) is present in the hypothalamic region outside sella turcica. In the middle line, no hypophyseal infundibulum (arrow head) is present. In the T2-weighted coronal section; b) septum pellucidum is present in the middle line (arrow). In the coronal section fat-suppressed; c) hypoplastic bilateral optic nerves are demonstrated (arrows)

A 5.5-year-old girl was referred to our clinic with complaints of short stature, strabismus and decreased vision. She was born at term with a birth weight of 3200 g and had mild retardation in the early stages of development of gross motor skills. There was no consanguinity between the parents. Her three siblings were stillborn. On physical examination, her body weight was 21 kg [+0.47 standard deviation score (SDS)], height was 98.5 cm (-3.04 SDS) and body mass index was 21.6 kg/m2 (+2.32 SDS). She had truncal obesity and external strabismus in the left eye. The patient had no dysmorphic features and showed no pathologic neurological finding. Her pubertal development was consistent with Tanner stage I. Complete blood count, liver and renal function tests and routine urine analysis were within normal limits. Thyroid function tests and prolactin level were also normal [thyroid-stimulating hormone (TSH) 1.69 mIU/mL (normal= 0.4-5), free thyroxine (fT4) 1.41 ng/mL (normal=0.8-1.9)]. Bone age was consistent with 3.5 years (Greulich-Pyle). A GH stimulation test using insulin showed a peak GH level of 0.12 ng/mL and a peak cortisol level of 19.4 µg/dL. In the ophthalmological examination, bilateral optic nerve hypoplasia was reported. Cranial MRI revealed an intact septum pellucidum with bilateral optic hypoplasia, ectopic neurohypophysis and agenesis of the hypophyseal infundibulum (Figure 1). The bilateral optic hypoplasia and findings of hypopituitarism led to a diagnosis of SOD. Echocardiography and speech audiometry revealed normal findings. Because of the cranial MRI findings, a second GH stimulation test was not performed and based on the results of the first test which showed an inadequate GH response to hypoglycaemia, recombinant human GH treatment in a dose of 25 µg/kg/d was initiated. At the end of the first year of treatment, the patient’s growth velocity reached 12.2 cm/year and her bone age was consistent with 4.5 years. During follow-up, the patient developed hypothyroidism [(TSH= 4.61 mIU/mL (normal= 0.4-5), fT4= 0.78 ng/mL (normal= 0.8-1.9)]. The results of thyrotropin-releasing hormone (TRH) stimulation test was consistent with central hypothyroidism (peak TSH at 60 minutes 10.8 

mIU/mL), thus L-T4 replacement therapy at a dose of 2 µg/kg/d was initiated. At the age of 8.25 years, the patient was noted to show onset of breast development (Tanner stage II). In subsequent months, she underwent a rapid progression of puberty and only six months later, she presented with menarche. At this time, her bone age had advanced to age 12 years. Her basal follicle-stimulating hormone, luteinizing hormone and estradiol levels were 6.47 mIU/mL, 7 mIU/mL and <20 pg/mL, respectively. These clinical and laboratory findings showed that our patient had an accelerated and rapidly progressive CP. Gonadotropin-releasing hormone analogue treatment (triptorelin acetate 3.75 mg/IM every 28 days) was initiated. 

## DISCUSSION

Clinical signs of SOD are quite variable. In addition to midline brain abnormalities, dysmorphic findings pertaining to the cranium (microphthalmia, anophthalmia, etc.) and to the musculoskeletal system can also be observed (3,6,7,8). Currently different definitions are being used for SOD. Some authors designate this rare disorder as SOD complex which presents with endocrine, central nervous system and skeletal abnormalities (7). Riedel et al (9) have suggested that the association of optic nerve hypoplasia and anterior pituitary hormone deficiency should be defined as an SOD variant when a normal septum pellucidum is present. According to this description, the current case can be defined as an SOD variant, because she showed signs of hypopituitarism and bilateral optic nerve hypoplasia without any midline brain defects.

Frequently, the first clinical sign in cases with SOD is decreased vision due to optic nerve hypoplasia. Neurological signs, which may mimic hemiparesis and epilepsy, may also be observed. Cases with sensorineural hearing loss, anosmia, cardiac and esophagus abnormalities, as well as cases with minimal or no cortical disorders have also been reported (7,10,11). Signorini et al (4) evaluated 17 patients with SOD and reported that they all had optic nerve hypoplasia; 14/17 had midline brain defects; 9/17 had anterior pituitary hormone deficiencies; and 7/17 had cortical developmental malformations. In our patient, neurological signs, hearing loss, cardiac pathology, or any other dysmorphic signs accompanying anterior pituitary hormone deficiencies and bilateral optic nerve hypoplasia were not present. Our patient was also found to have ectopic neurohypophysis, which has been reported in 50% of cases with SOD syndrome (11). 

Clinical signs of pituitary hormone deficiencies, which range from isolated single hormone deficiency to panhypopituitarism, can be observed in SOD cases (2). Signs of hormone deficiency may not be present initially and may develop over time (6). GH deficiency, adrenal insufficiency, hypothyroidism, pubertal disorders (early or late puberty) and inappropriate antidiuretic hormone syndrome have all been reported in patients with SOD (8). Hypophyseal endocrine disorders are reported in 65.9% of the patients and the most frequent one is GH deficiency (40.9%) (2). Morishima et al (7) reported that all three cardinal signs were observed in 30% of patients with SOD, while hypopituitarism and septum pellucidum agenesis were detected in 62% and 60% of the patients, respectively. Atapattu et al (2) reported that diabetes insipidus (DI) accompanied anterior pituitary hormone deficiencies in 21.5% of patients, while DI was an isolated sign in only two cases. Quvrier et al (8) detected cerebral malformation (septum pellucidum agenesis) in 25% of SOD patients diagnosed with bilateral optic nerve hypoplasia. In the current case, isolated GH deficiency was found to exist at presentation and central hypothyroidism developed during the follow-up. Although bilateral optic nerve hypoplasia was detected in MRI, no anatomic abnormality was observed in either septum pellucidum or in the corpus callosum.

While delayed puberty has been frequently encountered in SOD patients, occurrence of CPP associated with SOD is less often. Hanna et al (9) reported early and rapidly progressive puberty in three and delayed puberty in four out of their 13 SOD patients. Husemann et al (10) found precocious puberty in one out of five patients with SOD. Ladjouze et al (11) reported CPP in four patients (2 were males), who were being followed with a diagnosis of SOD and GH deficiency. This relatively rare concomitance of GH deficiency and CPP has been more frequently reported in SOD patients in whom SOD was accompanied by arachnoid cysts (5,9,10,11,12). Although the mechanism of CPP development in SOD patients is not known, it has been proposed that it might develop due to the effects of hypothalamic lesions (11). According to KIGS (Pfizer International Growth Study) database, 0.6% of patients receiving GH therapy developed CPP, without any obvious reason (13). Although it has been suggested that the underlying reason for CPP in patients with SOD might be GH therapy, precocious puberty has also been reported in SOD patients who were not receiving GH therapy (14). Obesity due to hypothalamic dysfunction can also develop in SOD patients during the follow-up. Based on this information, Ladjouze et al (11) proposed that the underlying cause of CPP in patients with a developmental defect of the hypothalamic pituitary area might be hypothalamic dysfunction. Our patient was found to show an accelerated pubertal development during her follow-up, while she was receiving GH treatment, onset of breast development rapidly followed by occurrence of menarche. Although it was suggested that one of the causes of CPP in these patients might be GH therapy, as marked weight gain was observed during the follow-up, we tend to agree with those who suggest that precocious puberty or accelerated progression of puberty might develop secondary to hypothalamic dysfunction. In these patients, acceleration in bone age and growth velocity before pubertal signs are reported. Therefore, it has been proposed that this condition has frequently delayed the diagnosis of GH deficiency. On the other hand, when GH therapy is initiated, the increase in growth rate may be interpreted in favor of efficacy of GH therapy and CPP may be misdiagnosed in these patients (11). In our patient also, before onset of pubertal signs, the acceleration in growth rate and improvement in bone age were first interpreted as a GH effect. 

In conclusion, with this case report, we would like to emphasize that (i) patients diagnosed with bilateral optic nerve hypoplasia should be investigated for anterior pituitary hormone deficiencies, (ii) inappropriate acceleration of growth velocity and rapid improvement in bone age may be predictive for CPP development during the follow-up of SOD patients receiving GH therapy.
